# Dynamic changes in peripheral blood lymphocyte subset counts and functions in patients with diffuse large B cell lymphoma during chemotherapy

**DOI:** 10.1186/s12935-021-01978-w

**Published:** 2021-05-27

**Authors:** Hongyan Hou, Ying Luo, Guoxing Tang, Bo Zhang, Renren Ouyang, Ting Wang, Min Huang, Shiji Wu, Dengju Li, Feng Wang

**Affiliations:** 1 Department of Laboratory Medicine, Tongji Hospital, Tongji Medical College, Huazhong University of Science and Technology, Jiefang Road 1095, Wuhan, 430030 China; 2 Department of Hematology, Tongji Hospital, Tongji Medical College, Huazhong University of Science and Technology, Wuhan, China

**Keywords:** Diffuse large B cell lymphoma, Lymphocyte subsets, CD4^+^ T cells, CD8^+^ T cells, NK cells

## Abstract

**Background:**

This study aimed to analyze the lymphocyte subsets, their activities and their dynamic changes during immunochemotherapy in patients newly diagnosed with diffuse large B cell lymphoma (DLBCL).

**Methods:**

Patients with DLBCL (n = 33) were included in the present study. Their peripheral lymphocyte subsets, phenotypes and functions were detected using flow cytometry. The dynamic results of lymphocyte activities were available for 18 patients.

**Results:**

Compared with healthy controls (HCs), the counts of CD3^+^, CD4^+^, and CD8^+^ T cells as well as those NK cells decreased in patients newly diagnosed with DLBCL, mainly attributed to patients with high risk of prognosis assessed by International Prognostic Index (IPI) score. Lymphocyte counts didn’t present significant difference between high risk (IPI scores 3–5) and low risk patients (IPI scores 0–2), but CD4^+^ T cells and CD8^+^ T cells expressed higher levels of CD28 and HLA-DR, respectively, in patients with IPI score ranging from 3 to 5. Patients at high risk harbored higher percentage of regulatory T cells (Tregs), and their CD4^+^ and CD8^+^ T cells produced lower levels of IFN-γ, reflecting an impaired cellular immune response. The dynamic changes of lymphocyte numbers and functions during treatment were further investigated. Total counts of CD3^+^, CD4^+^, CD8^+^ T and NK cells progressively decreased because of the cytotoxicity of chemotherapy and then gradually recovered after six cycles treatment (rituximab combined with cyclophosphamide, doxorubicin, vincristine and prednisone, R-CHOP). The functions of CD4^+^ and CD8^+^ T cells recovered by the end of two cycles R-CHOP treatment, although NK cell function was not significantly affected throughout treatment. These results suggest that the counts and functions of lymphocytes are significantly decreased in patients with DLBCL, particularly those of CD4^+^ and CD8^+^ T cells.

**Conclusions:**

The absolute counts and functions of CD4^+^, CD8^+^ T cells, which were significantly lower in patients with DLBCL, gradually recovered after effective treatment. Therefore, combined detection of T cell counts and functions are critically important for administering effective personalized immunotherapy as well as for identifying new prognostic markers or DLBCL.

**Supplementary Information:**

The online version contains supplementary material available at 10.1186/s12935-021-01978-w.

## Background

Diffuse large B-cell lymphoma (DLBCL) is the most common subtype of non-Hodgkin's lymphoma (NHL), accounting for approximately 40% of lymphoid malignancies [1]. DLBCL is clinically, pathologically, and molecularly heterogeneous; and the responses of patients to therapy and their survival times are highly variable. The International Prognostic Index (IPI) is widely used to identify patients at high risk of death [2]. However, combined treatment with the anti-CD20 monoclonal antibody rituximab (R), cyclophosphamide, doxorubicin, vincristine and prednisone (CHOP) dramatically improves the treatment outcomes of patients with DLBCL regardless of IPI score and pathological type [3, 4]. Moreover, one-third of patients will be refractory or develop a relapse after R-CHOP treatment [5], indicating that potential risk factors must be identified to develop more effective therapies.

The host’s immune system exerts critical anticancer effects, and its dysfunction contributes to pathogenesis and disease progression [6, 7]. Lymphocytopenia serves as a marker of poor prognostic during the initial stages of NHL, HL and relapsed DLBCL [8]. Early recovery of lymphocytes after autologous and allogeneic stem cell transplantation serves as a significant predictor of lymphoma control and a patient’s survival. The absolute lymphocyte counts and subsets reflected systemic immunity and predict the prognosis of patients with DLBCL. Furthermore, the success of cancer immunotherapy shows the importance of analysis of the host’s systematic immune response to assess the outcome of DLBCL [9, 10].

The types and numbers of specific T cell subsets that infiltrate B-cell lymphomas are associated with relapse and survival [11]. CD4^+^ T cells are generally considered beneficial and lower numbers of these cells are associated with a poor response to treatment and unfavorable prognosis of patients with DLBCL [11–13]. In contrast, elevated numbers of infiltrating cytotoxicity CD8^+^ T cells are associated with good outcomes of patients with B cell lymphomas [14]. Regulatory T cells (Tregs) suppress the functions of other immune cells and may correlate with prognostic factors of high risk [15]. Furthermore, natural killer (NK) cells play a critical role in innate immune surveillance, and a deficiency in NK cells is a potential marker of the severity of disease [8]. Although previous studies have reported the relationships between the lymphocyte numbers and lymphomas, further investigations are required to evaluate immune status through determining the numbers and functions of peripheral-blood lymphocytes.

Here we prospectively determined the numbers, phenotypes, and functions of peripheral CD4^+^ T, CD8^+^ T cells, B cells, and NK cells upon diagnosis according to IPI scores. We next determined the dynamic changes in lymphocyte subsets and activities in response to treatment with R-CHOP. Further evaluation of the immune responses of patients with DLBCL is critically important for efforts to identify new prognostic markers and to develop individualized immunotherapies.

## Methods

### Patients

This was a retrospective study, approved by the ethical committee of Tongji hospital, Tongji Medical College, Huazhong University of Science and Technology. From January 2018 to October 2019, 33 newly diagnostic DLBCL patients were recruited, including 13 females and 20 males. 18 patients were classified as stage I/II and 15 patients were classified as stage III/IV according to the Ann Arbor classification system. In the patients receiving treatment, each cycle was period was 20 days and 18 patients accompanied at least six cycles of R-CHOP treatment. In addition, 33 age and sex matched healthy controls (HCs) were also recruited and determined by interview and physical examination. The exclusive criteria include HIV and HCV positive. Peripheral blood for detection of lymphocyte subsets, phenotype and function was collected at each of following time-points: T0, newly diagnosis; T1, after the accomplishment of two cycles’ treatment; T2, after four cycles’ treatment; T3, after six cycles’ treatment. All of the subjects gave written informed consent.

### TBNK lymphocyte counting

The percentages and absolute numbers of CD4^+^ T cells, CD8^+^ T cells, B cells, and NK cells were determined using TruCOUNT tubes and BD Multitest 6-color TBNK Reagent Kit (BD Biosciences) according to the manufacturer’s instructions. In brief, 50 μL of whole blood was labeled with 6-color TBNK Ab cocktail for 15 min in room temperature. After adding 450 μL of FACS lysing solution, samples were analyzed with FACSCanto flow cytometer using FACSCanto clinical software (BD Biosciences).

### Lymphocyte phenotype analysis

The following monoclonal antibodies and regents were purchased from BD Biosciences and added to 100 µl of whole blood. The panel of antibodies in tube 1 were anti-CD45-PerCP (652,803), anti-CD3-APC-H7 (663,490), anti-CD4-V450 (560,345), anti-CD8-PE/Cy7 (335,822), anti-CD28-PE (340,046), anti-HLA-DR-APC (652,809). The panel of antibodies in tube 2 were anti-CD45-PerCP (652,803), anti-CD3-APC-H7 (663,490), anti-CD4-V450 (560,345), antiCD45RA-FITC (662,840), and anti-CD45RO-PE (663,530), anti-CD25-APC (662,525) and anti-CD127-PE/Cy (560,822). The phenotype of NK cells were detected using anti-CD45-PerCP (652,803), anti-CD3-TITC (349,201), anti-CD56-PE/Cy7 (652,825) and CD69-PE (557,050). Isotype controls with irrelevant specificities were included as negative controls. All of these cell suspensions were incubated for 20 min at room temperature. After lysing red blood cells with lysing solution (349,202), the cells were washed and re-suspended in 200 μl of PBS. The cells were then analyzed with FACSCanto flow cytometer.

### Lymphocyte function analysis

The evaluation of lymphocyte function should be conducted before chemotherapy at each time point. PMA/ionomycin-stimulated lymphocyte function assay was performed as described in our previous studies [16, 17]. The procedures were described as follows: 1) 100 µl of whole blood was diluted with 400 µl of IMDM medium; 2) the diluted whole blood was stimulated with Leukocyte Activation Cocktail (Becton Dickinson GolgiPlug) for 4 h; 3) the cells were stained with the following monoclonal antibodies anti-CD45-PerCP (652,803), anti-CD3-TITC (349,201), anti-CD4-APC/H7 (341,115), anti-CD56-PE/Cy7 (652,825), and anti-CD8-PE (340,046), followed by fixation and permeabilized (554,722), and staining with intracellular anti-IFN-γ-APC antibody (554,702). 4) the cells were analyzed with FACSCanto flow cytometer.

### Statistical analysis

The results are presented as median ± standard deviation (SD). The differences of markers among different groups were compared by analysis of variance (ANOVA). Statistical analyses were performed using GraphPad Prism version 6. Statistical significance was determined as *p* < 0.05.

## Results

The present study included 33 patients diagnosed with DLBCL, including 13 (39.4%) females and 20 (60.6%) males, median age 56 years (25^th^-75^th^ quartiles, range 36.5–67.5 years). Patients included were classified with stages I/II (n = 18, 54.5%) and III/IV (n = 15, 45.5%) according to the Ann Arbor classification system. The IPI scores of 23 and 10 patients ranged from 0 to 2 and 3 to 5, respectively. Patients’ characteristics and laboratory test results of are shown in Table 1.

**Table 1:** Baseline characteristics of patients

Characteristics	Values
Patients	33
Median age, years (range)	56 (36.5–67.5)
Sex	
Female	13 (39.4%)
Male	20 (60.6%)
Ann Arbor stage	
I–II	18 (54.5%)
III–IV	15 (45.5%)
Laboratory results	
LDH (U/L)	442.2 ± 399.3
Neutrophiles (× 10^9^ per L)	3.933 ± 2.077
Lymphocytes (× 10^9^ per L)	1.523 ± 1.981
Hemoglobin (g/L)	110.7 ± 28.93
Platelet (× 10^9^ per L)	185.2 ± 108.3
IPI score	
0–1	11 (33.3%)
2	12 (36.4%)
3	6 (18.2%)
4–5	4 (12.1%)

Data are presented as mean ± SD or numbers (%)

**Fig. 1:**
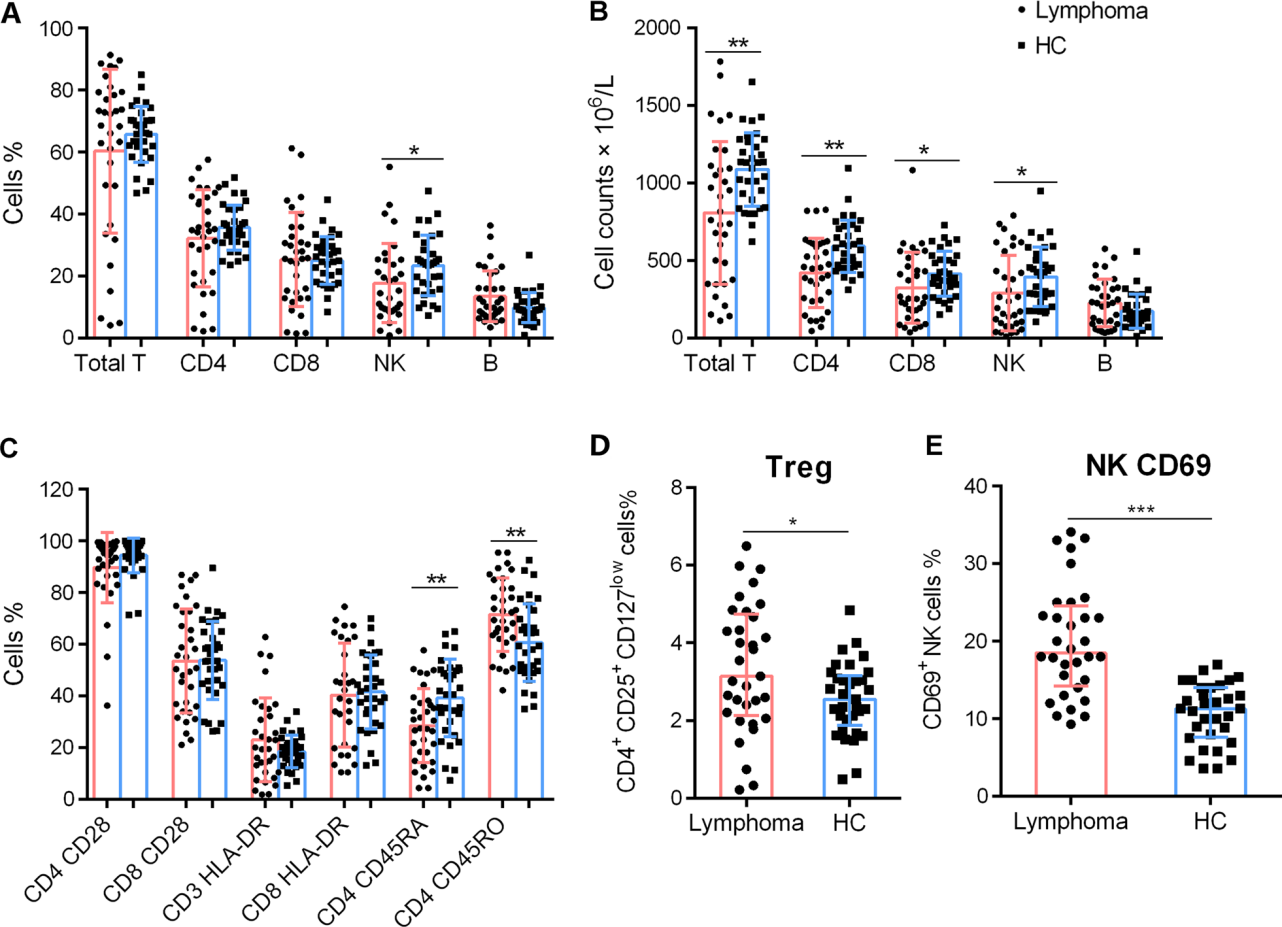
The lymphocyte subsets and immunophenotypic characteristics. Circulating lymphocytes in patients newly diagnosed with DLBCL and healthy controls (HCs) were analyzed using flow cytometry. **A**, **B** The percentages and absolute numbers of T cells, B cells and NK cells in different groups are shown in bar graphs (mean ± SD). **C** The expression of CD28 on CD4^+^ and CD8^+^ T cells, the expression of HLA-DR on CD3^+^ and CD8^+^ T cells, and the expression of CD45RO and CD45RA on CD4^+^ T cells in different groups are shown in bar graphs (mean ± SD). **D** The percentages of Tregs (CD4^+^ CD25^+^ CD127^low^) in lymphocytes are shown in bar graphs (mean ± SD). (E) The percentages of CD69 in NK cells are shown in bar graphs (mean ± SD). *p < 0.05, **p < 0.01, ***p < 0.001

Analysis of lymphocyte subsets in peripheral blood revealed that decreased proportions of NK cells were present in patients with DLBCL at diagnosis compared with those of HCs, although there were no significant differences in the frequencies of CD3^+^, CD4^+^, and CD8^+^ T cells and B cells (Fig. 1A). Accordingly, the absolute numbers of CD3^+^, CD4^+^, CD8^+^ T cells and NK cells were significantly lower in patients with DLBCL compared with those of HCs, although the numbers of B cells were similar (Fig. 1B). The templates used for the analysis of lymphocyte phenotypes in patients with DLBCL were shown in Additional file 1: Figure S1. The results show that the levels of the co-stimulatory molecule CD28 and the immune activation marker HLA-DR expressed by CD4^+^ T and CD8^+^ T cells were not significantly different (Fig. 1C). Decreased percentages of naïve T cells (CD4^+^/CD45RA^+^/CD45RO^−^) and elevated percentages of memory T cells (CD4^+^/CD45RA^−^/CD45RO^+^) were observed in patients with DLBCL (Fig. 1C). Furthermore, the proportions of Tregs (CD4^+^/CD25^+^/CD127^low^) and CD69^+^ NK cells were higher in patients with DLBCL compared with those of HCs (Fig. 1D, E).

**Fig. 2:**
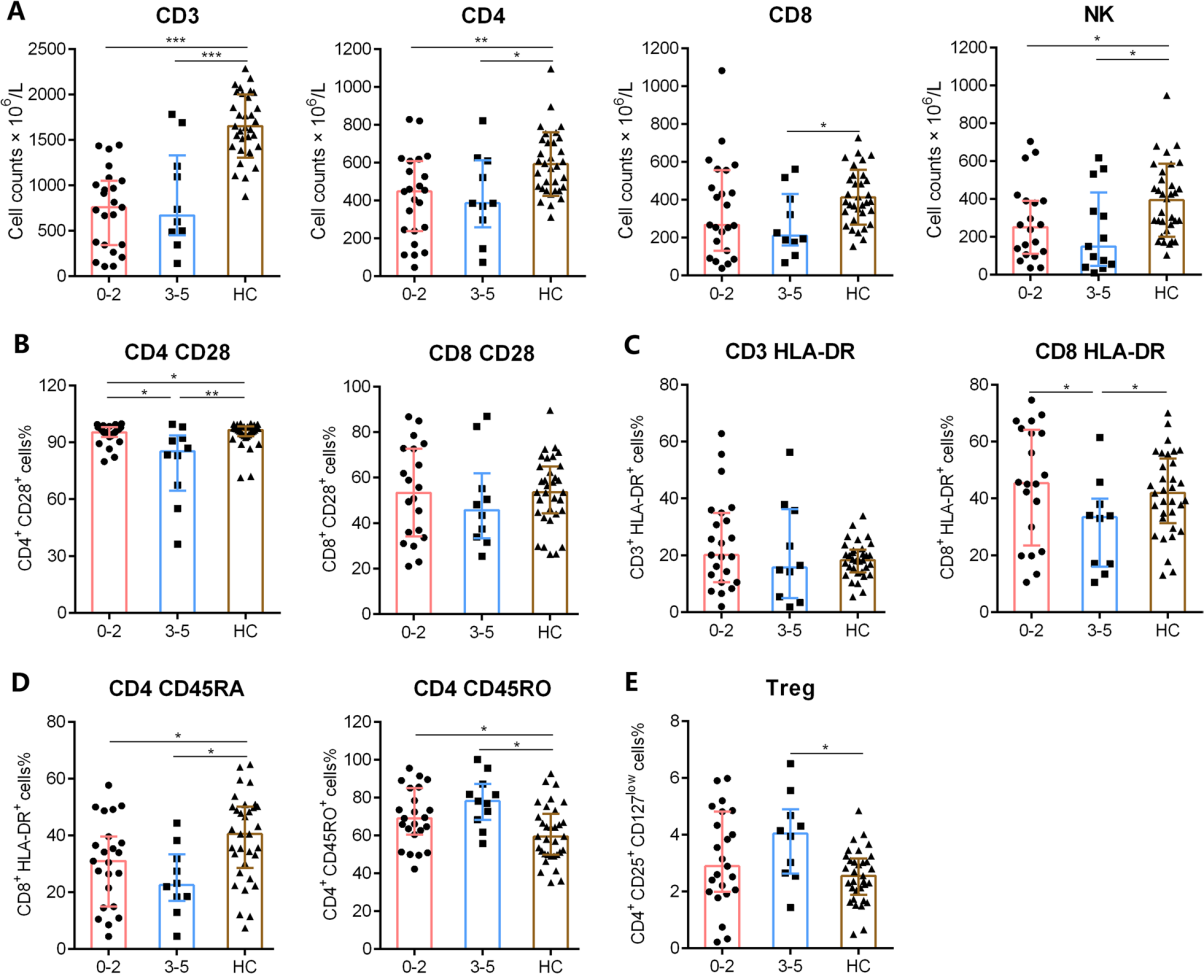
The subsets and phenotypes of lymphocytes in patients with DLBCL according to IPI scores. Patients with DLBCL were stratified into low- to low-mediate-risk (IPI scores 0–2) and mediate-high- to high-risk (IPI scores 3–5) groups. The absolute counts of CD3^+^ T, CD4^+^ T, CD8^+^ T cells and NK cells (**A**), the percentages of CD28^+^ cells in CD4^+^ and CD8^+^ T cells (**B**), the percentages of HLA-DR^+^ cells in CD3^+^ T and CD8^+^ T cells (**C**), the percentages of CD45RO^+^ and CD45RA^+^ cells in CD4^+^ T cells (**D**) and percentages of Tregs in lymphocytes are shown in different groups (mean ± SD). *p < 0.05, **p < 0.01, ***p < 0.001

The IPI score, which is the main prognostic factor for DLBCL, was used to stratify patients into low- to low-middle-risk (IPI scores 0–2), high-middle to high-risk (IPI scores 3–5) groups. The absolute counts of CD3^+^, CD4^+^ T cells and those of NK cells were lower in the low-risk (IPI scores 0–2) and high-risk (IPI scores 3–5) groups compared with those of HCs. The numbers of CD8^+^ T cells were relatively lower only in high-risk patients. However, there were no significant differences between lymphocyte subsets of the two groups (Fig. 2A). The levels of CD28 expressed by CD4^+^ T cells were lower in low-risk patients (IPI scores 0–2) compared with those of high-risk patients (IPI scores 3–5); and the CD28 levels of both patient groups were lower compared with those of HCs (Fig. 2B). Lower percentages of HLA-DR expressed on CD8^+^ T cells were only observed in high-risk (IPI scores 3–5) patients (Fig. 2C). CD45RO and CD45RA levels differed among patients compared with those of HCs (Fig. 2D). Furthermore, increased percentages of Tregs were only observed in the high-risk group (Fig. 2E).

**Fig. 3:**
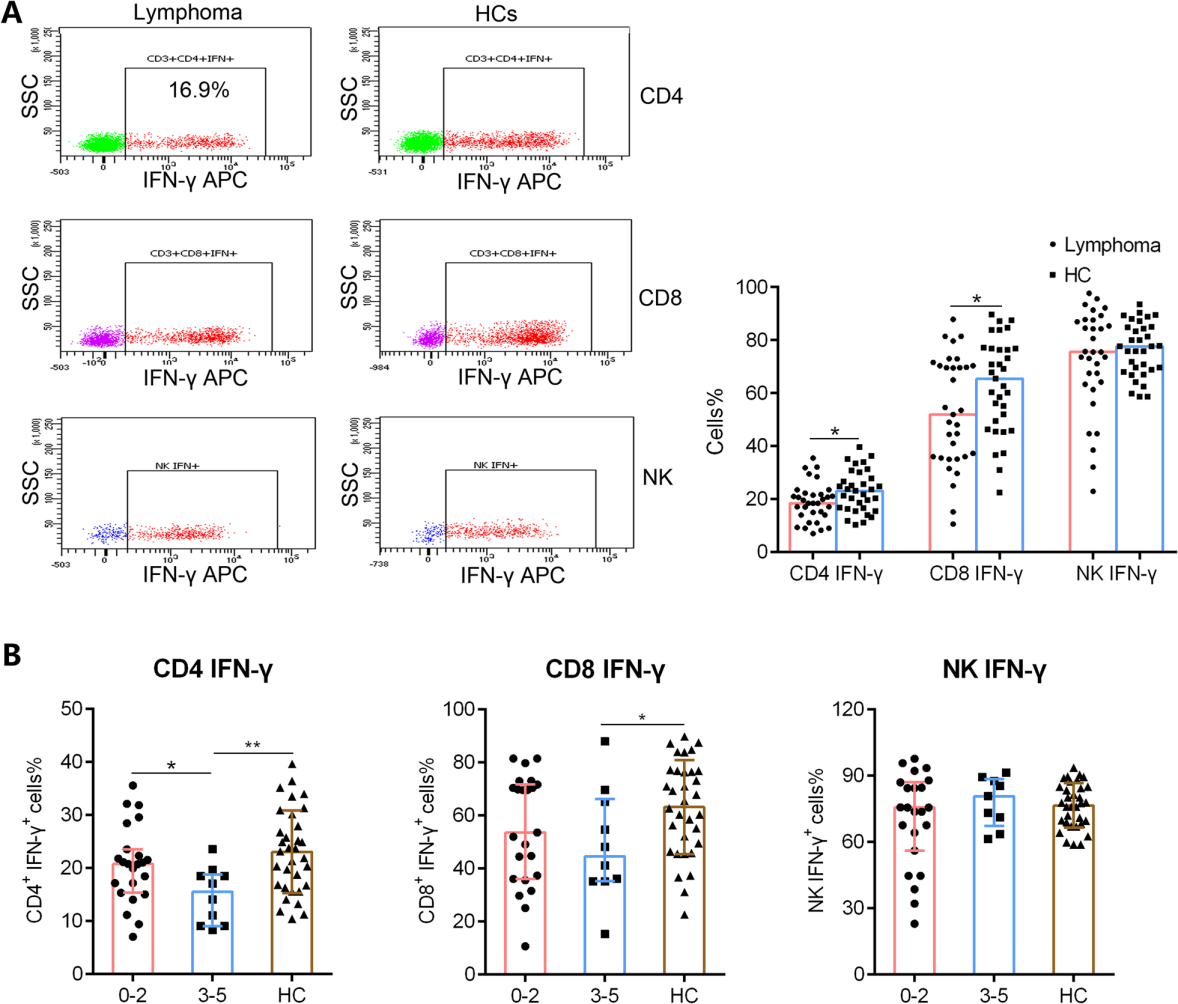
The IFN-γ producing ability of CD4^+^ T cells, CD8^+^ T cells, and NK cells. PMA/ionomycin-stimulated lymphocyte function assay was performed in patients with DLBCL and HCs. **A** Representative FACS dot plots showing the production of IFN-γ in CD4^+^ T cells, CD8^+^ T cells and NK cells. **B** The percentages of IFN-γ^+^ cells in CD4^+^ T, CD8^+^ T cells and NK cells from patients with lymphoma and HCs are shown in bar graphs (mean ± SD). **C** The percentages of IFN-γ^+^ cells in CD4^+^ T, CD8^+^ T cells and NK cells from patients with low- to low-mediate-risk (IPI scores 0–2) and mediate-high- to high-risk (IPI scores 3–5) are shown in bar graphs (mean ± SD)

The functions of lymphocytes were detected based on IFN-γ secretion assay upon PMA/ionomycin stimulation according to our previous studies [16–18]. An exhaustion pattern of T cells was observed, and patients’ CD4^+^ and CD8^+^ T cells showed lower capability of IFN-γ production compared with those of HCs, but the levels of IFN-γ produced by NK cells were not significantly lower (Fig. 3A). Further analysis revealed that the capability of IFN-γ production decreased significantly in high- risk (IPI scores 3–5) compared with those of HCs (Fig. 3B).

**Fig. 4:**
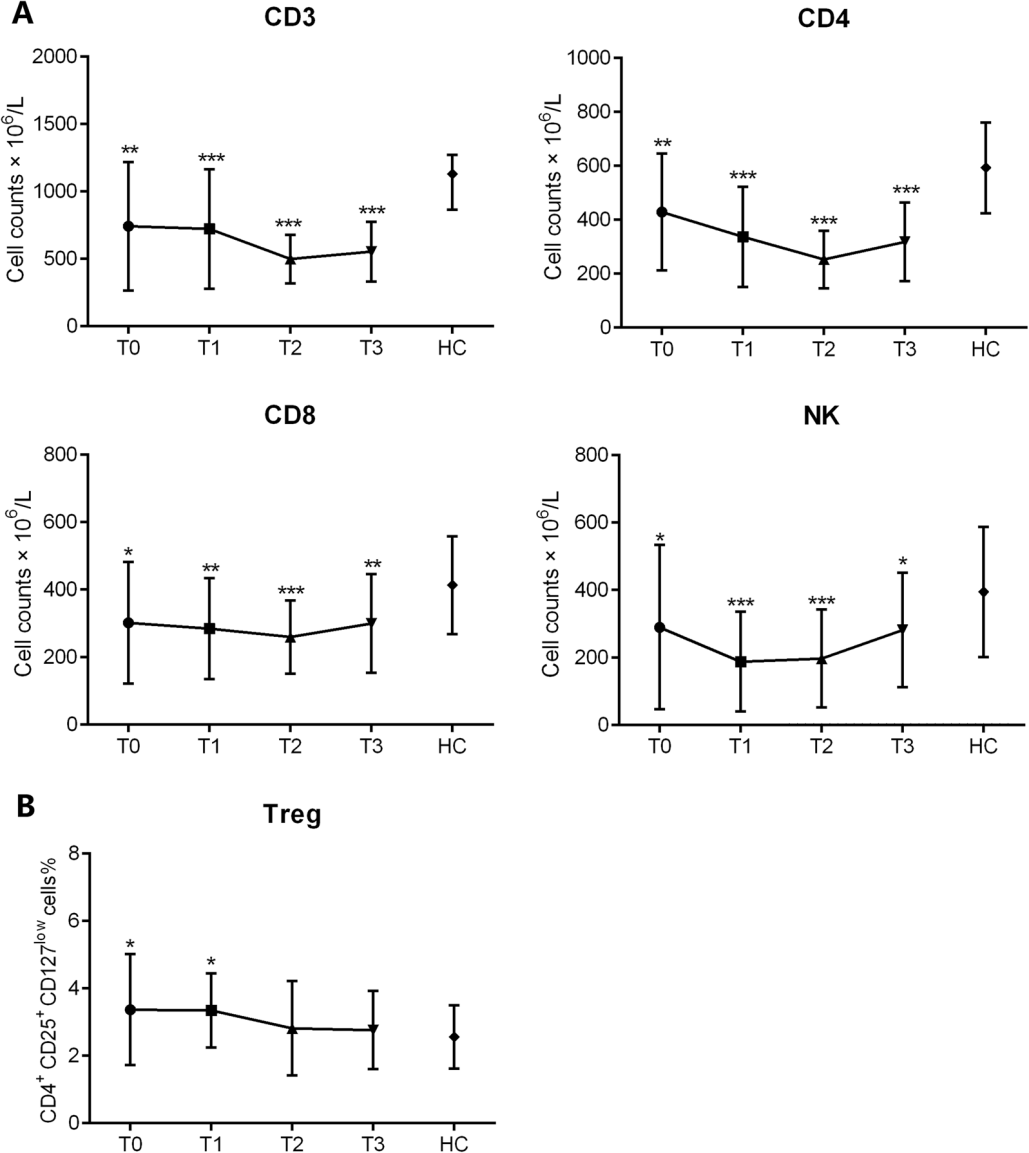
Dynamic changes of CD3^+^ T, CD4^+^ T, CD8^+^ T cells and NK cells and the percentages of Tregs. The lymphocyte subsets in patients with DLBCL were detected at T0, T1, T2 and T3, respectively. All *p* values were the results of comparison with HCs. (A) Absolute numbers of CD3^+^ T, CD4^+^ T, CD8^+^ T cells and NK cells are shown in bar graphs at different time points (mean ± SD). (B) Percentages of Tregs are shown at different time points

Eighteen patients who underwent ≥ 6 cycles’ treatment were analyzed to determine the dynamic changes in lymphocyte subsets and functions. The total counts of CD3^+^, CD4^+^, and CD8^+^ T cells progressively decreased during treatment, and reaching their lowest values after 4 cycles’ R-CHOP treatment (T2). Recovery of lymphocyte counts was detected during T3, although also significantly lower compared with those of HCs. The counts of NK cells significantly decreased at T1, and recovery was significantly during T3 (Fig. 4A). The percentages of Tregs relative to those of HCs were high at T1, although they decreased during T2 (Fig. 4B).

**Fig. 5:**
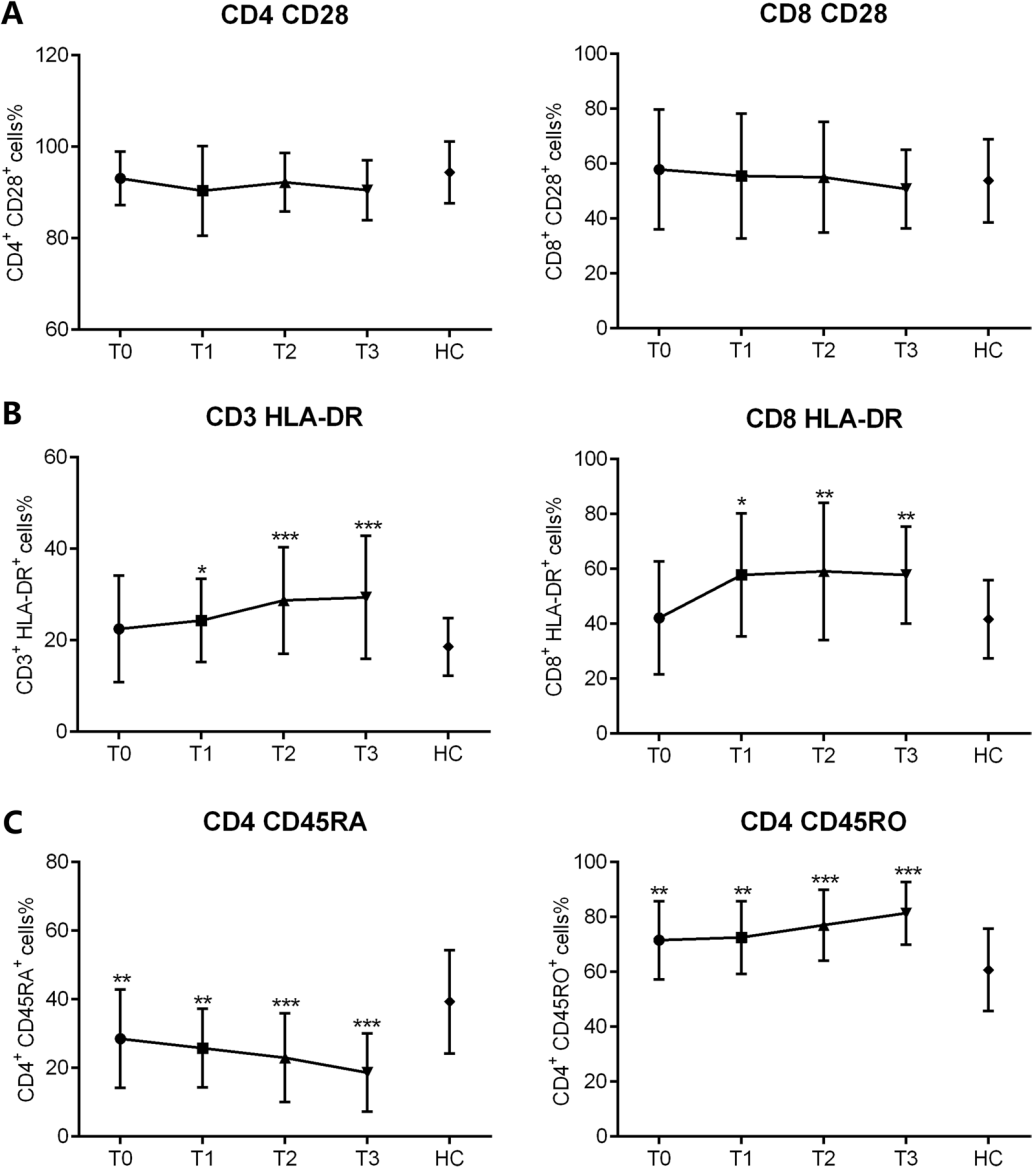
The dynamic changes of lymphocyte phenotypes. The expression of CD28, HLA-DR, CD45RO and CD45RA were detected at T0, T1, T2 and T3, respectively. The percentages of CD28^+^ cells in CD4^+^ and CD8^+^ T cells (**A**), the percentages of HLA-DR^+^ cells in CD3^+^ T and CD8^+^ T cells (**B**), and the percentages of CD45RO^+^ and CD45RA^+^ cells in CD4^+^ T cells (**C**) in patients during R-CHOP treatment are shown in bar graphs (mean ± SD)

**Fig. 6:**
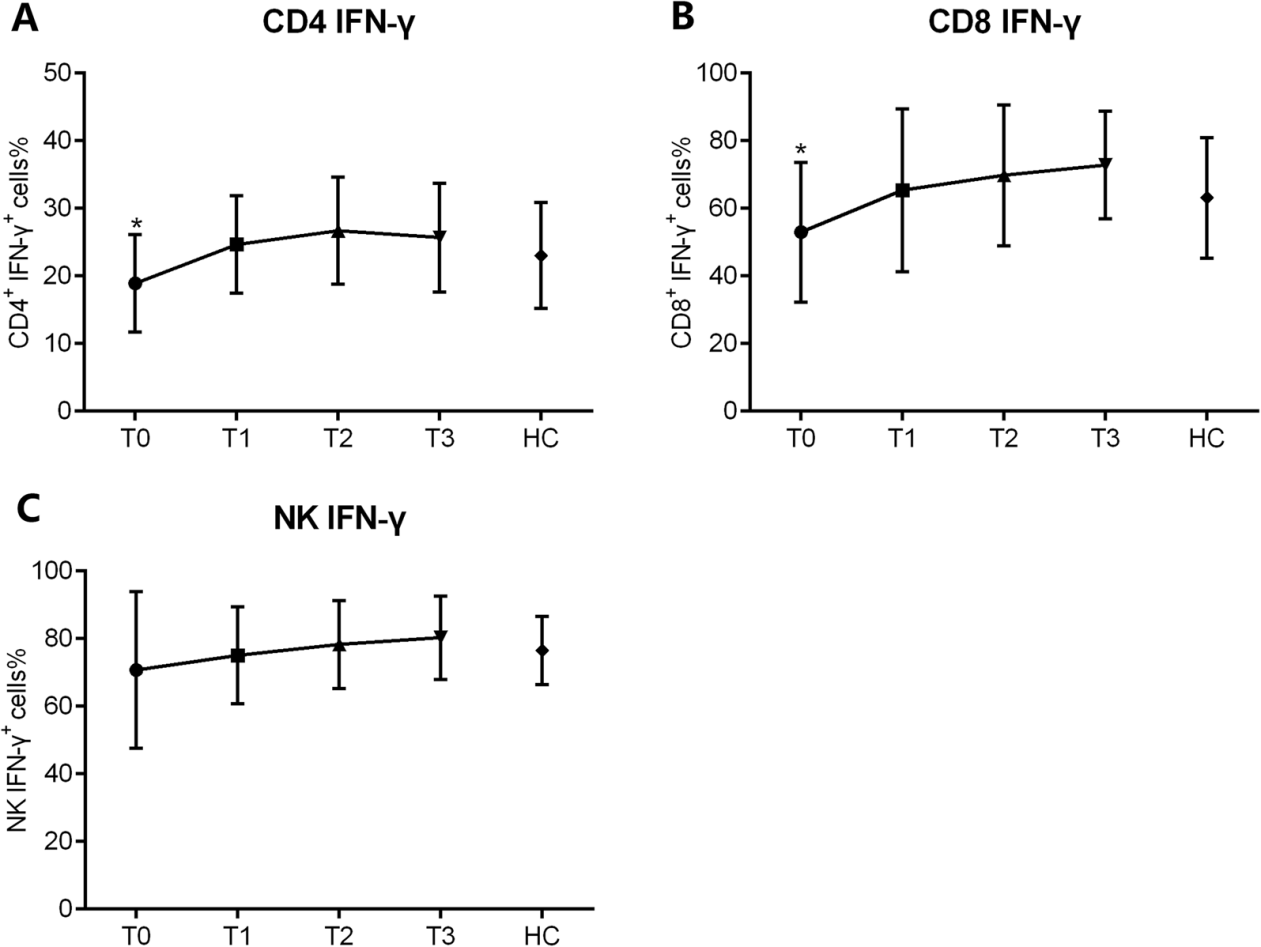
The dynamic changes of IFN-γ producing ability in CD4^+^ T cells, CD8^+^ T cells, and NK cells. The secretion capability of IFN-γ^+^ from CD4^+^ T, CD8^+^ T cells and NK cells were assessed at T0, T1, T2 and T3, respectively. Percentages of IFN-γ^+^ cells in CD4^+^ T (**A**), CD8^+^ T (**B**) and NK (**C**) cells are shown in bar graphs (mean ± SD)

When we next assessed the dynamics of activation lymphocytes, we found that the levels of CD28 expressed by CD4^+^ and CD8^+^ T cells were relatively, although those of HLA-DR gradually increased during T1 (Fig. 5A, B). The percentages of naïve CD45RA^+^ CD4^+^ T cells decreased, whereas those of memory CD45RO^+^ CD4^+^ T cells increased during R-CHOP treatment (Fig. 5C). Consistent with T cell phenotypes, the functions of CD4^+^ and CD8^+^ cells gradually increased during T1, but not that of NK cells (Fig. 6A, B, C). Continuous clinical monitoring revealed that 12 (66.7%) patients achieved complete remission (CR). The mean absolute numbers of CD3^+^ T (937.8 ± 513.0 vs. 375.1 ± 218.5, *p* = 0.022), CD4^+^ T (417.7 ± 205.1 vs. 223.8 ± 114.9, *p* = 0.041), CD8^+^ T (327.8 ± 160.3 vs. 159.0 ± 90.8, *p* = 0.017) and NK cells (320.4 ± 197.4 vs. 257.5 ± 197.2, *p* = 0.468) were higher in patients who achieved a CR compared with those who did not.

## Discussion

R-CHOP serves as first-line therapy of DLBCL because it significantly lengthens patient survival. Rituximab eradicates malignant cells through complement-dependent cytotoxicity (CDC), and antibody-dependent cell-mediated cytotoxicity (ADCC) and may induce a robust anti-tumor effect through activation of the immune system [19]. NK cells and T cells mediate this process, which is enhanced through and the activation of CD4^+^ T cells. In the present study, we reveal alterations of lymphocyte subsets and functions in patients with DLBCL. Specifically, we found that lower counts of CD3^+^, CD4^+^, CD8^+^ T cells and NK cells and higher percentages of Tregs are present in such patients upon initial diagnosis compared with those of HCs, suggesting depressed immune activity. For example, low T cell counts serve as an important risk factor and an important predictor of progression-free survival (PFS) and overall survival (OS) [9, 13].

We show here that CD4^+^ T cells were generated from naïve cells (CD45RO^−^ CD45RA^+^) to an effector/memory (CD45RO^+^ CD45RA^−^) phenotype in patients with DLBCL, although the functional marker HLA-DR and the levels of costimulatory marker CD28 expressed by CD4^+^ and CD8^+^ T cells were not significantly elevated. NK are major population of cells in peripheral blood that kill tumor cells, and NK cell counts are associated with disease outcomes [20, 21]. Furthermore, a deficiency in NK cells may correlate with the progression and severity of disease [20]. Therefore, low numbers of lymphocyte subsets are significant markers of immune dysfunction and disease prognosis.

Patients with lower T cell counts have a poor prognosis [22, 23]. We therefore analyzed the correlation of lymphocyte subsets with IPI scores. For this purpose, we stratified patients with DLBCL into a low- to low-mediate-risk group (IPI scores 0–2) and mediate-high- to high-risk groups (IPI scores 3–5). Although the absolute counts of lymphocyte subsets were lower in the mediate-high- to high-risk group compared with those of low to low-mediate-risk group (IPI scores 0–2), but the differences were not significantly. These findings are consistent with those of previous study showing that lymphocyte subset counts are not associated with bone marrow involvement or tumor burden [20]; however, further investigation of larger population are required. Decrease expression of CD28 by CD4^+^ T cells and active cytotoxicity of CD8^+^ T cells was observed in patients with high IPI scores. These results suggest that the decrease in lymphocyte numbers and active phenotypes was a secondary manifestation of DLBCL, independent of the IPI score. This may be explained by the inability of IPI score to identify a poor prognostic group with less than a 50% chance of survival after the administration of rituximab [24]. Therefore, broader explorations of the host’s immune system may identify more informative indicators of the prognosis of patients with lymphoma.

In the present study, we observed decreased IFN-γ production by CD4^+^ and CD8^+^ T cells, revealing an impaired cellular immune response of patients with DLBCL. CD8^+^ T cells are the major effector cells that mediate antitumor immunity, although antigen-specific CD4^+^T cells contribute to the activation of antigen-presenting dendritic cells and the secretion of cytokines [25]. Therapies that reverse immunosuppression and immune dysfunction may prove to be most effective. Therefore, the combined assessment of the numbers and functions of lymphocytes play a pivotal role in evaluation of the host’s immune status as well as for predicting the prognosis of patients with DLBCL who under immunotherapy.

The cytotoxic effects of chemotherapy reduce the numbers of CD3^+^, CD4^+^, CD8^+^ T cells and NK cells. Here, we found that the earliest recovery of lymphocytes occurred at the end of six cycles of R-CHOP treatment, although the counts of lymphocyte subsets in patients with DLBCL were still lower compared with those of HCs. In contrast, the percentage of Tregs decreased at the end of the second cycle R-CHOP treatment. Tregs suppress other immune cells and maintain immune tolerance, and higher percentages of Tregs correlate with shorter survival [15]. The absolute numbers of T cells and NK cells were higher in patients who achieved a CR compared with those who did not, suggesting that lymphocyte numbers may serve as informative therapeutic markers for predicting the response of patients to R-CHOP treatment. These results suggest that the absolute number of lymphocyte subsets is an important therapeutic marker for evaluating patients administered R-CHOP treatment. Furthermore, the percentage of HLA-DR^+^ T cells gradually increased, but not those of T cells expressed CD28. Lymphopenia and a dysregulated cytokine milieu are characteristic of DLBCL [26, 27], which is consistent with our present findings of T cell dysfunction. We further found that the T cells recovered their function at the end of 2 cycles of R-CHOP treatment. NK cells contribute to the innate immune surveillance of B cell lymphomas, and a deficiency in NK cell is associate with disease outcome [20]. Although impaired NK cell function occurred observed in cancers such as AML [28], it may be a minor effect on the progression of DLBCL. These results reflect generalized depressed immune activity, and the recovery of host immunity correlates with effective treatment. Further studies are required to analyze the correlation of lymphocyte function with the clinical features and OS.

This study had several limitations. First, lymphocytopenia occurs in NHL and HL, and thus lymphocyte counts and functions of other subsets of lymphomas other than DLBCL must be assessed in future studies. Second, the present results require validation through studies of more patients with lymphomas, particularly those that include comparisons of blood lymphocytes subpopulations with those of TILs. Third, the significant of combining lymphocyte counts, phenotypes and functions for predicting the prognosis of OS and PFS of patients with DLBCL requires further investigations.

## Conclusions

The numbers and functions of lymphocytes, mainly CD4^+^ and CD8^+^ T cells, were significantly decreased in DLBCL. The absolute numbers of lymphocytes were recovered gradually after 6 cycles treatment of R-CHOP, and the function recovered after 2 cycles treatment. These results show that changes in the lymphocyte-mediated immune response of patients with DLBCL occur during R-CHOP treatment and support the conclusion that systemic immune evaluation will significantly contribute to the development of innovative treatment to improve clinical prognosis.

## Supplementary Information

**Additional file 1**: **Figure S1**. The template for analysis of lymphocyte phenotypes by flow cytometry.

## Data Availability

Not applicable.

## Abbreviations

DLBCL: Diffuse large B cell lymphoma
HCs: Healthy controls
IPI: International Prognostic Index
R: Rituximab
CHOP: Cyclophosphamide, doxorubicin, vincristine and prednisone (CHOP)
NHL: Non-Hodgkin's lymphoma
Tregs: Regulatory T cells
NK: Natural killer
PFS: Progression-free survival
OS: Overall survival
