# Supplementary figures and images for: Dynamic changes in peripheral blood lymphocyte subset counts and functions in patients with diffuse large B cell lymphoma during chemotherapy
Source: Cancer Cell Int. 2021 May 27;21:282. doi: 10.1186/s12935-021-01978-w (PMC8162016; doi:10.1186/s12935-021-01978-w)

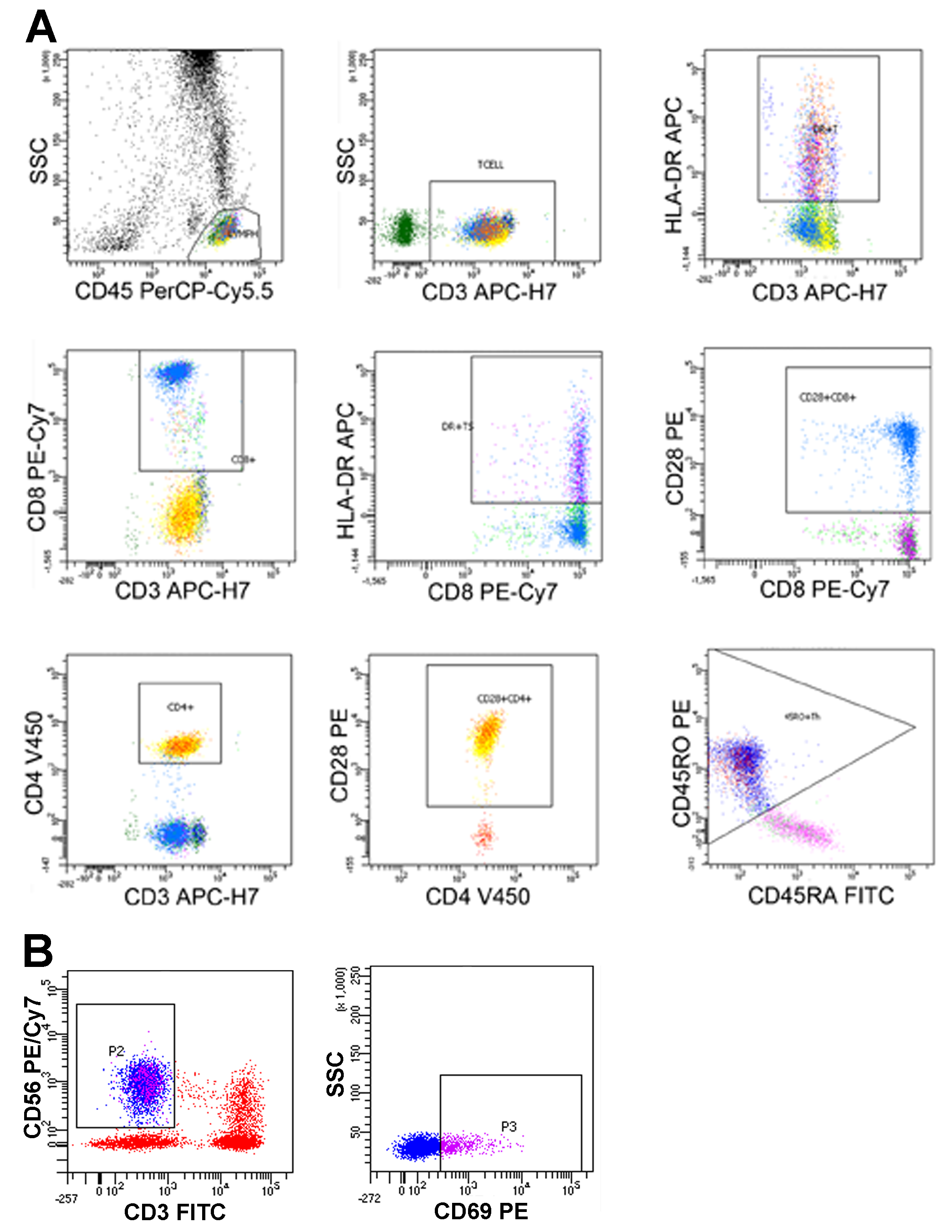

Supplement: Supplementary file 1 — Additional file 1: Figure S1. The template for analysis of lymphocyte phenotypes by flow cytometry. [file 12935_2021_1978_MOESM1_ESM.tif]
